# The Effect of Pungent and Tingling Compounds from *Piper nigrum* L. on Background K^+^ Currents

**DOI:** 10.3389/fphar.2017.00408

**Published:** 2017-06-26

**Authors:** Leopoldo R. Beltrán, Corinna Dawid, Madeline Beltrán, Janina Levermann, Sascha Titt, Sini Thomas, Viktoria Pürschel, Miriam Satalik, Günter Gisselmann, Thomas Hofmann, Hanns Hatt

**Affiliations:** ^1^Department of Cell Physiology, Ruhr-University-BochumBochum, Germany; ^2^Chair of Food Chemistry and Molecular Sensory Science, Technische Universität MünchenFreising, Germany; ^3^Department of Receptor Biochemistry, Ruhr-University-BochumBochum, Germany

**Keywords:** *Piper nigrum* L., piperine, pungency, tingling, taste, KCNK channels, trigeminal neurons

## Abstract

Black peppercorns (*Piper nigrum* L.) elicit a pungent and tingling oral impression. Their pungency is partially explained by the agonist activity of some of their active principles, especially piperine, on TRP channels. However, we recently showed that piperine, as well as other pungent compounds, also possess a marked effect on two-pore domain (KCNK, K_2P_) K^+^ channels. Members of this family play a key role in maintaining the resting membrane potential of excitable cells. Interestingly, tingling compounds have been shown to induce neuronal excitation by inhibiting KCNK channels. We addressed the question of whether it was plausible that KCNK channels could constitute a physiologically relevant target for the sensory active compounds present in black peppercorns. Because previous studies have demonstrated that mouse trigeminal neurons respond to several pungent compounds, to which humans are also sensitive, we used a primary culture of mouse trigeminal neurons to investigate whether the effect of piperine on these cell types could also be mediated by KCNK channels. We observed that even in the presence of classical TRP-antagonists, piperine was still able to activate a fraction of trigeminal neurons. Furthermore, our results showed that piperine is capable of inducing neuronal depolarization by a mechanism that does not require extracellular Na^+^ or Ca^2+^. This depolarization was mediated by the inhibition of a background K^+^ conductance, most likely corresponding to the KCNK channels of the TASK subfamily. We then performed a screening with 12 other pungent and/or tingling chemosensates isolated from black peppercorns. These compounds were evaluated on *Xenopus laevis* oocytes expressing the human orthologues of KCNK3, KNCK9 and KCNK18, which we previously showed to be inhibited by piperine. Remarkably, almost all of the isolated chemosensates inhibited the basal activity of hKCNK3, with 1-(octadeca-2*E*,4*E*,13/12*Z*-trienoyl)pyrrolidine acting as one of the most potent natural blockers for hKCNK3 found to date. Our results suggest that KCNK channels, especially KCNK3, are likely to play a complementary role to TRP channels in the complex orosensory impression elicited by black peppercorns, while they also help to expand the pharmacological knowledge of KCNK channels.

## Introduction

Black pepper (*Piper nigrum* L.) is one of the most consumed spices worldwide. While its alluring aroma is due to a group of odor-active key volatiles (Dunkel et al., 2014), peppercorns have been attracting consumers for over centuries due to its characteristic pungent and tingling orosensory impressions.

**FIGURE 1:**
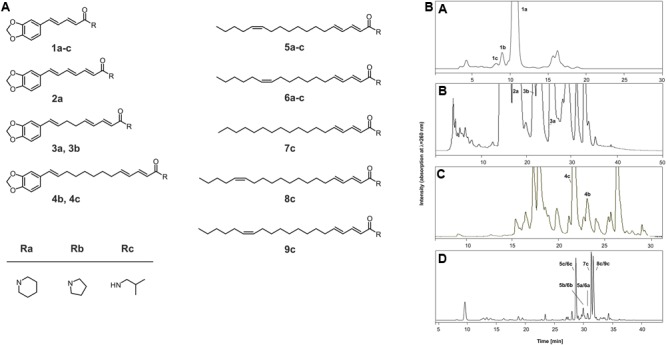
**(A)** The chemical structures of pungent and tingling compounds **1–9c** in black pepper and its pungent^p^ and tingling^t^ threshold concentrations, as reported by Dawid et al. (2012): piperine **(1a)** [3.0 nmol/cm^2p^], piperyline **(1b)** [5.1 nmol/cm^2p^], piperlonguminine **(1c)** [10.4 nmol/cm^2p^], piperettine **(2a)** [5.2 nmol/cm^2p^], dehydropipernonaline **(3a)** [152.1 nmol/cm^2p^], 1-[1-oxo-9(3,4-methylenedioxyphenyl)-2*E*,4*E*,8*E*-nonatrienyl]-pyrrolidine **(3b)**, brachyamide A **(4b)**, guineensine **(4c)** [810.1 nmol/cm^2p^], 1-(octadeca*-*2*E*,4*E*,13*Z*-trienyl)piperidine **(5a)**, 1-(octadeca*-2E,4E,13Z*-trienyl)pyrrolidine **(5b)**, (2*E*,4*E*,13*Z*)-*N*-isobutyl-octadeca-2,4,13-trienamide **(5c)** [540.5 nmol/cm^2p^; 2162.1 nmol/cm^2t^], 1-(octadeca*-*2*E*,4*E*,12*Z*-trienyl)piperidine **(6a)**, 1-(octadeca*-*2*E*,4*E*,12*Z*-trienyl)pyrrolidine **(6b)**, (2*E*,4*E*,12*Z*)-*N*-isobutyl-octadeca-2,4,12-trienamide **(6c)** [540.5 nmol/cm^2p^; 2162.1 nmol/cm^2t^], (2*E*,4*E*)-*N*-isobutyl-octadeca-2,4-dienamide **(7c)** [763.0 nmol/cm^2p^], (2*E*,4*E*,15*Z*)-*N*-isobutyl-eicosa-2,4,15-trienamide **(8c)** [741.2 nmol/cm^2p^; 1482.3 nmol/cm^2t^], (2*E*,4*E*,14*Z*)-*N*-isobutyl-eicosa-2,4,14-trienamide **(9c)** [741.2 nmol/cm^2p^; 1482.3 nmol/cm^2t^]. The compounds **5c/6c** and **8c/9c** were tasted as binary mixtures. **(B)** A RP-HPLC chromatogram (λ = 260 nm) of SPE fraction A **(A)**, SPE fraction B **(B)** SPE fraction C **(C)**, and SPE fraction D **(D)** showing the pepper amides **1a–9c**.

Early research on the pungent principles of black peppercorns (Oersted, 1820; Landenburg and Schaltz, 1894) led to the isolation and identification of piperine **(1a)** (40-50 mg/100 g peppercorn) (**Figure 1A**), which is the predominate amide and major chemosensate of black peppercorns (Freist, 1991; Srinivasan, 2007; Friedman et al., 2008). Recently, the application of a sensomics approach to black peppercorns, including taste dilution analysis (TDA) followed by liquid chromatography-tandem mass spectrometry (LC-MS/MS), ultra-performance liquid chromatography-time of flight-mass spectrometry (UPLC-Tof-MS) and one-dimensional/two-dimensional nuclear magnetic resonance spectroscopy (1D/2D NMR) experiments as well as synthesis, led to the structural determination of 25 key pungent and tingling amides (Dawid et al., 2012). In particular, two chemical classes of non-volatiles have been identified with pungent and tingling organoleptic properties, namely the the piperine-type analogs decorated with a piperonal moiety as found in piperlonguminine (**1c**), piperyline (**1b**) or brachyamide A (**4b**), and the unsaturated, long-chain fatty acid amides such as 1-(octadeca-2*E*,4*E*,13/12*Z*-trienyl)piperidine (**5a**/**6a**), 1-(octadeca-2*E*,4*E*,13/12*Z*-trienyl)pyrrolidine (**5b**/**6b**) or (2*E*,4*E*,13/12*Z*)-*N*-isobutyl-octadeca-2,4,13-trienamide (**5c**/**6c**), respectively. Sensory studies by means of a modified half-tongue test using filter paper rectangles as the vehicle confirmed that the lowest threshold concentration for pungency was presented by piperine (**1a**, 3.0 nmol/cm^2^), followed by piperyline (**1b**, 5.0 nmol/cm^2^) and piperlonguminine (**1c**, 10.4 nmol/cm^2^), (Dawid et al., 2012). Previous studies have found that ethanol causes a potentiation of vanilloid receptor-1 activity (Trevisani et al., 2002), and the purified test compounds, which showed low water solubility, were not sensorially evaluated in hydroalcoholic solutions but on filter paper vehicles (1 cm × 2 cm) put on the tongue surface (Dawid et al., 2012). Interestingly, while piperine analogs exclusively exhibited a clear pungent sensory profile, both a pungent impression as well as a long-lasting tingling sensation at higher concentrations were observed for a group of conjugated 2,4-dienoic acid amides with an additional *cis*-configured double bound as found in 1-(octadeca*-*2*E*,4*E*,13*Z*-trienyl)piperidine (**5a**), 1-(octadeca*-*2*E*,4*E*,13*Z*-trienyl)pyrrolidine (**5b**), (2*E*,4*E*,13*Z*)-*N*-isobutyl-octadeca-2,4,13-trienamide (**5c**) and 1-(octadeca*-*2*E*,4*E*,12*Z*-trienyl)piperidine (**6a**) (Dawid et al., 2012).

While sensory evaluation demonstrated, the structural features causing the pungent and tingling impression of these pepper amides, the precise molecular and cellular mechanism by which such impressions are elicited is not fully understood. To elicit these characteristic impressions, sensory active compound from black peppercorns activate trigeminal neurons, which are primary sensory neurons whose bodies reside in the trigeminal ganglion (Viana, 2011). These cells are specialized in conveying information regarding different sensory events, such as light pressure, heat, cold, as well as helping to provide information regarding the chemical characteristics of the environment (Xu et al., 2010; Viana, 2011). The functionally diverse populations of trigeminal neurons have been classified according to their size, the neurotransmitters they release, their “current signature” (Xu et al., 2010), as well as their pharmacological profile (Spehr et al., 2004). To be able to react to chemical stimuli trigeminal neurons rely on chemosensitive proteins expressed on their membranes, mostly ion channels (Gerhold and Bautista, 2009; Viana, 2011). The most notable among these are TRP channels, which are polymodal ion channels that can be activated by a plethora of chemically diverse compounds, especially pungent sensing ones (Caterina et al., 1997; Ramsey et al., 2006). Studies form the past 10 years have shown that the TRP channels TRPV1 (McNamara et al., 2005) and TRPA1 (Okumura et al., 2010) constitute a target for piperine. However, although important, this substance alone does not completely explain the complex orosensory impression elicited by black peppercorns. In 2010, Okumura et al. reported that other constituents from *Piper nigrum* also activated TRPV1, as well as TRPA1; however, further research showed that the human recognition threshold concentrations for many of these substances did not correlate with the data obtained from heterologously expressed TRP channels (Okumura et al., 2010; Dawid et al., 2012). Additionally, no mechanism explaining the tingling impression of black peppercorns has been proposed to date. This raised the possibility that additional receptors in addition may be required for eliciting the characteristic impression of black peppercorns.

Recently, we have shown that piperine inhibits the basal activity of three two-pore domain (KCNK, K_2P_) channels, which are also expressed in trigeminal neurons of both human and rodent: KCNK3 (TASK-1, K_2P_3), KCNK9 (TASK-3, K_2P_9), and KCNK18 (TRESK, K_2P_18) (Beltrán et al., 2013; Manteniotis et al., 2013; Flegel et al., 2015). These constitutively open, potassium selective channels are important for maintaining the resting membrane potential of excitable cells (Lotshaw, 2007; Enyedi and Czirják, 2010). Inhibition (or deletion) of KCNK channels has been shown to increase neuronal sensitivity toward a given stimulus, or even to induce neuronal excitation (Bautista et al., 2008; Noël et al., 2009). We previously showed that *Xenopus laevis* oocytes expressing hKNCK3 were strongly depolarized by the application of piperine (Beltrán et al., 2013). Interestingly, this substance presented an IC_50_ value at the same range of its reported EC_50_ value for TRPV1 (McNamara et al., 2005; Beltrán et al., 2013). Moreover, they have been shown to be targets for the tingling chemosensate hydroxy-α-sanshool (Bautista et al., 2008). Additionally, next generation sequencing experiments have shown that KCNK3, KCNK9 and KCNK18 are expressed in human and mouse trigeminal ganglia, with KCNK3 showing the second highest expression, after the non-heterologously expressing KCNK12, in humans (Flegel et al., 2015). These studies led us to hypothesize that KCNK channels could constitute a target for the above-mentioned sensory active compounds isolated from black peppercorns.

## Materials and Methods

### Chemicals

The following compounds were obtained commercially: formic acid, ethanol (Merck, Darmstadt, Germany); solvents were of HPLC grade (Merck Darmstadt, Germany). Water used for chromatography was purified by means of a Milli-Q water advantage A 10 water system (Millipore, Molsheim, France). The black peppercorn samples were purchased from the German retail market. The Capsaicin, Capsazepine, ethylene glycol tetraacetic acid (EGTA), dimethyl sulfoxide (DMSO), HC-030031, and piperine were purchased from Sigma–Aldrich (Steinheim, Germany). The stock solutions of 100 mM were diluted in DMSO and were made for all the employed sensory active chemical compounds. These were stored at –20°C. The aliquots were then diluted to obtain the desired concentrations.

### Solvent Extraction of Black Pepper

Following the protocol reported recently (Dawid et al., 2012) a portion of black peppercorn (100 g) was frozen in liquid nitrogen, ground in a laboratory blender (Analysenmühle A10, Ika Labortechnik, Germany), and then extracted with ethanol (6 × 25 mL) at room temperature upon ultrasonification (10 min). After filtration (MN 6151/4, 150 mm), the combined organic extract was separated from ethanol in vacuum at 30°C, freeze-dried for 48 h, and then kept at –20°C until used (yield: 3.16 g/100 g).

### Solid Phase Extraction

An aliquot (800 mg) of the lyophilized ethanol extract isolated from black peppercorns was suspended in methanol/water (60/40, v/v; 20 mL) and applied onto the top of a Strata C18-E SPE cartridge (10 g/60 mL; Phenomenex, Aschaffenburg, Germany) preconditioned with methanol (60 mL), followed by methanol/water (60/40, v/v; 100 mL). Using a vacuum extraction box (J. T. Baker, Philipsburgh, NJ, United States) separation was performed by flushing the cartridge with a sequence of methanol/water mixtures (100 mL each) to give fraction A (70/30, v/v; yield: 35.4%), fraction B (80/20, v/v; yield: 29.8%), fraction C (90/10, v/v; yield: 6.0%), and finally fraction D (100/0, v/v; yield: 3.6%). The individual fractions were collected, separated from solvent in vacuum, the residues were taken up in water, freeze-dried twice, and were then kept at –20°C prior to use.

### Preparative Isolation and Identification of Pepper Amides in Fraction A

First, fraction A was dissolved in a mixture of methanol/water (70/30, v/v, 2mL/50 mg) and, after membrane filtration, was injected onto a 250 mm × 21.2 mm i.d, 5 μm, Microsorb-MV C18 column (Varian, Darmstadt, Germany) equipped with a guard column of the same type operated with a flow rate of 18 mL/min. Using 0.1% formic acid in water (v/v) as solvent A and methanol (v/v) as solvent B, chromatography was performed with the following gradient: 0 min, 50% B; 10 min, 50% B; 20 min, 70% B; 30 min, 80% B; 35 min, 100% B; 40 min, 100% B; 45 min, 50% B; 55 min, 50% B. Monitoring the effluent at 260 nm the peaks were collected individually in several runs, the eluates of the corresponding fractions were combined, and the purity of each fraction (>98%) was checked by means of analytical RP-HPLC. After removing the solvent in vacuum and freeze-drying twice, single compounds were directly used for cell experiments as well as for structure determination by means of UV-Vis, LC-MS/MS, UPLC-TOF-MS, and 1D/2D NMR experiments, while the fractions containing mixtures of individual substances were purified by re-chromatography using semi-preparative HPLC. Thereby, the pungent chemosensates piperlonguminine (**1c**), piperyline (**1b**), and piperine (**1a**) were identified in HPLC fractions given in **Figure 1B (A)**. The analytical details of the structure determination, including the comprehensive set of MS and NMR data were published previously (Dawid et al., 2012).

### Preparative Isolation and Identification of Pepper Amides in Fraction B

After membrane filtration, a solution of an aliquot of SPE fraction B in methanol/water (80/20, v/v, 2 mL/50 mg) was separated by means of semi-preparative HPLC using a 250 mm × 10.0 mm i.d, 5 μm, HyperClone ODS (C18) column (Phenomenex, Aschaffenburg, Germany) equipped with a guard column of the same type with gradient elution at a flow rate of 5 mL/min. Chromatography was performed using 0.1% formic acid in water (v/v) as solvent A and methanol (v/v) as solvent B: 0 min, 70% B; 25 min, 75% B; 40 min, 100% B; 42 min, 100% B; 45 min, 70% B; 50 min, 70% B. The effluent containing the targeted pepper amides [monitored at 260 nm; cf. **Figure 1B (B)**] was collected from several separate HPLC runs, combined, freed from solvent in vacuum, and freeze-dried twice. After checking the purity of each fraction (>98%) by means of analytical RP-HPLC, single compounds were directly analyzed by means of LC-MS and NMR spectroscopy, while the fractions containing mixtures of individual substances were purified by re-chromatography using semi-preparative HPLC. For example, substance **2a** was further purified by re-chromatography using the same column and solvents as detailed above. Thereby, an isocratic solvent gradient (5 mL/min; 70% B) was used for chromatography. Prior to injection, the column was equilibrated for 10 min at the starting conditions. The details of the NMR and MS experiments demonstrating the chemical identity of the pepper amides piperettine (**2a**), 1-[1-oxo-9(3,4-methylenedioxyphenyl)-2*E*,4*E*,8*E*-nonatrienyl]-pyrrolidine (**3b**), and dehydropipernonaline (**3a**) isolated from HPLC fractions given in **Figure 1B (B)** are published previously (Dawid et al., 2012).

### Preparative Isolation and Identification of Pepper Amides in Fraction C

In addition, SPE fraction C was dissolved in a mixture of methanol/water (80/20, v/v, 2 mL/50 mg) and, after membrane filtration, was sub-fractionated by means of preparative HPLC on a 250 mm × 21.2 mm i.d, 5 μm, Microsorb-MV C18 column (Varian, Darmstadt, Germany). Solvent A was 0.1% formic acid in water (v/v) and solvent B was methanol (v/v). The LC program consisted of a binary gradient (flow rate: 20 mL/min; λ = 260 nm): 0 min, 70% B; 5 min, 80% B; 35 min, 90% B; 40 min, 100% B; 42 min, 100% B; 50 min, 70% B. The peaks were collected individually in several runs, and the eluates of the corresponding fractions [cf. **Figure 1B (C)**] were combined. After determining the purity of each fraction by means of analytical RP-HPLC to be more than 98%, HPLC-DAD, HPLC-MS/MS, UPLC-TOF-MS and NMR analysis led to the identification of guineensine (**4c**) and brachyamide A (**4b**) as reported previously (Dawid et al., 2012).

### Preparative Isolation and Identification of Unsaturated Long-Chain Fatty Acid Amides in Fraction D

Aliquots of fraction D were dissolved in methanol/water (90/10, v/v, 2mL/50 mg) and, then, after membrane filtration, were injected onto a 250 mm × 21.2 mm i.d, 5 μm, Microsorb-MV C18 column (Varian, Darmstadt, Germany) equipped with a guard column of the same type. Using 0.1% formic acid in water (v/v) as solvent A and methanol as solvent B, chromatography was performed with the following gradient (flow rate: 18 mL/min; λ = 260 nm): 0 min, 75% B; 30 min, 100% B; 35 min, 100% B; 38 min, 75% B; 45 min, 75% B. The effluent was separated into 5 subfractions, namely, 1-(octadeca*-*2*E*,4*E*,13/12*Z*-trienyl)piperidine (**5a/6a**), 1-(octadeca*-*2*E*,4*E*,13/12*Z*-trienyl)pyrrolidine (**5b/6b**), (2*E*,4*E*,13/12*Z*)-*N*-isobutyl-octadeca-2,4,13/12-trienamide (**5c/6c**), (2*E*,4*E*)-*N*-isobutyl-octadeca-2,4-dienamide (**7c**), (2*E*,4*E*,15/14*Z*)-*N*-isobutyl-eicosa-2,4,15/14-trienamide (**8c/9c**), which were collected individually in several runs [cf. **Figure 1B (D)**]. To obtain the target compounds the solvent was removed in vacuum at 38°C, followed by lyophilization. Spectroscopic data (UV-Vis, LC-MS/MS, UPLC-TOF-MS, and NMR) and chromatographic data (RP-HPLC) were identical to the data obtained previously (Dawid et al., 2012).

### High Performance Liquid Chromatography (HPLC)

The HPLC system (Jasco, Groß-Umstadt, Germany) consisted of two PU-2087 Plus pumps, a 3-line DG-2080-53 degasser, an AS-2055 Plus autosampler, a 7725i type Rheodyne injection valve (Rheodyne, Bensheim, Germany), and a MD-2010 Plus diode array detector monitoring the effluent in a range between 220 and 500 nm. The data acquisition was performed by means of Chrompass 1.8.6.1 (Jasco, Groß-Umstadt, Germany).

### Molecular Biology

Human KCNK3 (TASK-1, K_2P_3.1) cloned into the expression vector pIRES-CD8 was a gift from Dr. Fabrice Duprat (Institut National de la Santé et de la Recherche Médicale, Antipolis, France). Human KCNK18 (hTRESK, K_2P_18.1), cloned into the plasmid pEXO, was a gift from Dr. P. Enyedi (Semmelweis University, Budapest, Hungary). These plasmids were linearized with the restriction enzymes *Bam*HI and *Xba*I, respectively, and then used as templates for *in vitro* transcription. Human KCNK9 (hTASK-3, K_2P_9.1) cloned into the expression vector pCR4-TOPO, was purchased from Imagenes (Berlin, Germany). The plasmid was linearized with the restriction enzyme *Ssp*I, to generate a linear cDNA template for *in vitro* transcription. mKCNK3 was isolated from mouse heart by standard procedures and inserted into pSGEM. mKCNK9 and mKCNK18 were kindly provided by Dr. D. Bayliss (University of Virginia, Charlottesville, VA, United States) and Prof. Dr. P. Enyedi (Semmelweis University, Budapest, Hungary). Both channels were subcloned into pSGEM. The pSGEM plasmids were subsequently linearized with the restriction enzyme *Pac*I. The capped RNAs were synthesized in the presence of the capping analog m7G(5′)ppp(5′)G using the AmpliCap-T7 Message Maker kit (Epicentre, Madison, WI, United States). The cRNA was dissolved in nuclease-free water to obtain a final concentration of 100 ng/μL.

### *Xenopus laevis* Oocytes Preparation

The expression in *Xenopus laevis* oocytes was essentially performed as previously described (Beltrán et al., 2013). Briefly, mature female *Xenopus laevis* frogs were anesthetized with 0.15% MS-222, and surgery was performed according to standard methods, where the extracted ovarian lobes were placed into Ca^2+^-free Barth solution containing collagenase type II (Worthington Biochemical Corporation, Lakewood, NJ, United States) and kept for 90 min on a shaker at 40 rpm (room temperature). Healthy stage V and VI oocytes were selected for injection with cRNA coding for the proteins of interest. Thirty nanoliters of cRNA (100 ng/μL) of each channel were injected using a Microinjector Micro 4 ^TM^ (World Precision Instruments). After injection, the oocytes were kept in ND96 (96 mM NaCl; 2 mM KCl; 1.8 mM CaCl_2_; 1 mM MgCl_2_; 2.5 mM Sodium pyruvate; 10 mM, HEPES [4-(2-hydroxyethyl)-1-piperazineethanesulfonic acid), pH 7.4]. The measurements were performed 24 to 72 h after injection. The oocytes were placed in a 100 μL chamber that was perfused with frog Ringer’s solution (115 mM NaCl; 2.5 mM KCl; 1.8 mM CaCl_2_, and 10 mM HEPES, pH 7.2). The substances were dissolved in this solution before being manually applied to the oocytes using an automatic pipette (Research pro, Eppendorf, Germany). The application time was usually three ramps (approximately 10 s). For recording, a Turbo Tec-03x (npi electronic GmbH, Tamm, Germany) amplifier was used, with an acquisition rate of 1000 Hz and a low pass filter of 20 Hz. Borosilicate glass capillaries were pulled with a Kopf vertical pipette puller. The pulled glass capillaries were filled with 3 M KCl.

### Two-Electrode Voltage Clamp Measurements

A ramp series protocol was used to evaluate the activity of KCNK channels as previously described (Beltrán et al., 2013). Briefly, voltage ramps from –100 mV to + 50 mV (0.21 mV/s) followed by a constant of +50 mV (150 ms) were applied at a time interval of 2 s. To evaluate the effect of a substance, we selected the three ramps that showed the maximal response to it, we then took the average of the current registered during the final 50 ms of the +50 mV constant. This value was then normalized to the average obtained from three ramps prior to the application. In most cases, the currents elicited by this voltage-clamp ramp series protocol were more than ten times larger than the background currents observed in uninjected oocytes, while also presenting the described outward rectification under physiological concentrations of intracellular and extracellular K^+^ (Goldstein et al., 2001; Enyedi and Czirják, 2010). Due to these characteristics, no leakage subtraction was considered necessary. Oocytes not fulfilling these characteristics were not considered for data analysis.

The data were collected using the *Cellworks Reader 3.7* software (npi instruments, Tamm, Germany) and analyzed with *Clampfit v10.2.0.14* (MDS Analytical Technologies, Sunnyvale, USA). The mean data at different concentrations were fitted using the Hill-1 equation (OriginPro v.9, OriginLab Corporation, Northampton, MA, United States): I = I_o_{[compound]ˆh/(IC_50_ˆh + [compound]ˆh)}, where h is the Hill slope, I is current amplitude obtained in the presence of a compound, normalized to current amplitudes obtained under control condition (I_o_). The data are expressed as the mean ± SEM. A paired *t*-test was used to evaluate the statistical significance of the results, where *P* < 0.05 was considered statistically significant and is marked with one star, *P* < 0.01 is marked with two stars, and *P* < 0.001 is marked with three stars.

### Primary Culture of Trigeminal Ganglia Neurons

Trigeminal ganglia neurons were isolated from Swiss CD-1 mice (Charles River Laboratories, Sulzfeld, Germany). All experiments involving animals were carried out in accordance with the European Union Community Council’s guidelines and approved by the competent state office of the Federal Land of Northrhine Westphalia (file number 87-51.04.2010.A180). At postnatal day 2–5, the mice were sacrificed by decapitation. The brain was removed, and both trigeminal ganglia were excised. The ganglia were washed in PBS+/+ (Invitrogen, Karlsruhe, Germany) and collected in Leibovitz Medium (L15, Invitrogen) on ice. The ganglia were transferred to Dulbecco’s modified essential medium (DMEM) containing 0.025% collagenase (type IA, Sigma–Aldrich). The minced ganglia were incubated for 45 min in a humidified atmosphere (37°C, 95% humidity, 6% CO_2_) before trituration. The cell suspension was centrifuged at 1000 rpm for 4 min at room temperature and the pellet was resuspended in F-12 medium (Invitrogen; 31331-D-MEM/F-12, GlutaMax^TM^) supplemented with 10% fetal bovine serum (FBS^TM^; Invitrogen), 200 U/mL penicillin and 200 U/mL streptomycin. The cell suspension (50 μL) was then plated on poly L-lysine (0.01%) coated glass coverslips (30 mm, Sarstedt, Nümbrecht, Germany), placed in cell culture dishes (35 mm) and kept in a humidified atmosphere (37°C, 95% humidity, 6% CO_2_) for 1 h. Subsequently, F-12 medium (2 mL) supplemented with FBS (10%), 200 U/mL penicillin and 200 U/mL streptomycin was added to each dish. The cells were kept in an incubator (37°C, 95% humidity, 6% CO_2_) until use.

### Calcium-Imaging Experiments

The trigeminal ganglion neurons were incubated with the Ca^2+^-sensitive reporter dye Fura-2/AM (Invitrogen, final concentration: 3 μM) 30–45 min before starting the experiment. The coverslips were placed under an inverted microscope equipped for ratiometric live cell imaging (Olympus IX71) with a 150-watt xenon arc lamp, a motorized fast change filter wheel illumination system (MT20, Olympus) for multiwavelength excitation. The loaded cells were viewed at 20× magnification and excited alternately at wavelengths of 340 nm and 380 nm. The emitted light (510 nm) was detected with a 12-bit 1376 × 1032-pixel charge-coupled device camera (F-View II, Olympus), and Cell^®^ imaging software (Olympus). The changes in intracellular calcium levels were calculated as the intensity ratio of f340/f380 [(E(510) at 340 nm illumination)/(E(510) at 380 nm illumination)]. The background intensity was subtracted from the fluorescent intensity changes. During an experiment, the cells were constantly superfused with extracellular solution, which could quickly be replaced by the desired test solution using a homemade 7-in-1 miniaturized metal tube application system. The viability and neuronal character of the cells were verified by a short (5 s) pulse of high potassium solution at the end of each experiment. The extracellular solution contained: 140 mM NaCl, 5 mM KCl, 2 mM CaCl_2_, 1 mM MgCl_2_, and 10 mM HEPES, pH 7.4. The high potassium solution contained: 150 mM KCl, 5 mM NaCl, 1 mM MgCl_2_, and 10 mM HEPES, pH 7.4.

### Electrophysiology in Trigeminal Neurons

The recordings were performed using the whole-cell configuration of the patch-clamp technique. The cells were maintained in an extracellular recording solution containing 140 mM NaCl, 5 mM KCl, 2 mM MgCl_2_, 2 mM CaCl_2_, 10 mM HEPES and 10 mM glucose, pH 7.4. The Ca^2+^ and Na^+^- free extracellular solution contained: 140 mM NMDG-Cl, 5 mM KCl, 2 mM MgCl_2_, 5 mM EGTA, 10 mM HEPES, pH 7.4. The patch electrodes were pulled from borosilicate glass (1.2 mm OD × 1.17 mm ID; Harvard apparatus, Edenbridge, Kent, United Kingdom) and fire polished to 2.5–4 MΩ tip resistance using a horizontal pipette puller (Zeitz Instruments, Munich, Germany) to obtain a patch of approximately 1–2 μm diameter. The pipette solution contained 140 mM KCl, 1 mM MgCl_2_, and 10 mM HEPES, pH 7.4. All recordings were performed at room temperature with an EPC7 amplifier (List-Medical Electronic, Darmstadt, Germany). The capacity and serial resistance were adjusted manually. The substances were applied via a custom-made manifold superfusion device. For recordings, a ramp series from –100 to 50 mV (0.214 mV/s) was used. The data were acquired using Pulse software (HEKA, Lambrecht, Germany) and analyzed with PulseFit (HEKA, Lambrecht, Germany). Unless otherwise stated, the data are expressed as the mean ± SEM. A paired *t*-test was used to evaluate the statistical significance of the results, where *P* < 0.05 was considered statistically significant and is marked with one star, *P* < 0.01 is marked with two stars, and *P* < 0.001 is marked with three stars.

## Results

**FIGURE 2:**
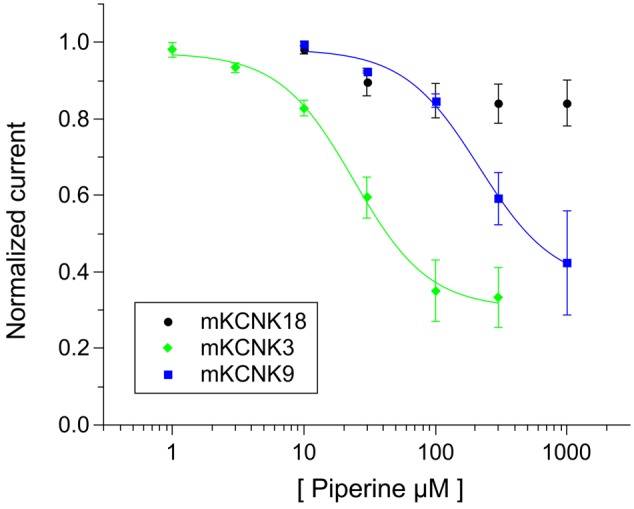
A dose-response relationship for the effect of piperine **(1a)** on *Xenopus* oocytes expressing the mouse orthologues for KCNK3, KCNK9, and KCNK18. *N* = 3–5 oocytes.

**FIGURE 3:**
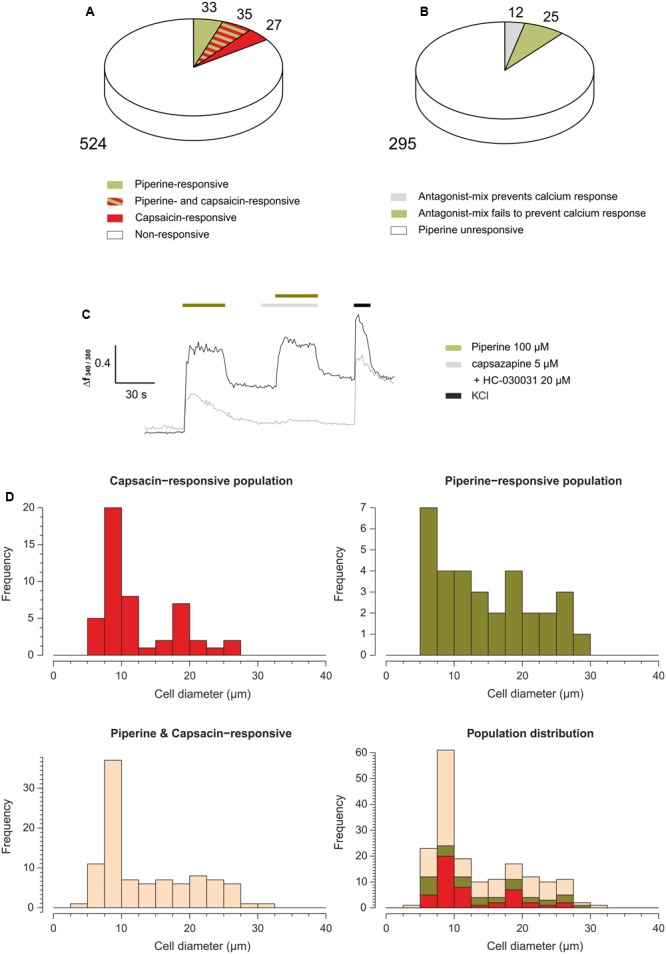
**(A)** Pie chart indicating the percentage of piperine- (100 μM) and/or capsaicin- (10 μM) responsive trigeminal neurons. *N* = 619 neurons from 23 separate experiments. **(B)** Pie chart indicating the percentages of piperine-unresponsive neurons (white slice), and piperine-responsive neurons (gray and green slices). The neurons that responded to a first application of piperine (100 μM) are subdivided into those in which a second response to piperine (100 μM) was prevented by preincubation and simultaneous co-application with an antagonist-mix consisting on 20 μM HC-030031 and 5 μM capsazepine (gray slice) and those in which the antagonist-mix failed to prevent a calcium response (green slice). *N* = 332 neurons from 13 separate experiments. **(C)** Representative Ca^2+^ imaging traces of two mouse trigeminal neurons subjected to the application of piperine 100 μM; capsazepine 5 μM and HC-030031 20 μM; piperine 100 μM, capsazepine 5 μM and HC-030031 20 μM; and KCl 150 mM. **(D)** Histogram of the distribution of mouse trigeminal neurons in their function to cell diameter and their responsiveness to capsaicin and piperine, *N* = 179 neurons from nine separate experiments.

We first investigated whether a KCNK-like conductance could be involved in the activation of mouse trigeminal neurons by piperine. Prior experiments on the effect of pungent compounds on KCNK channels were restricted to the *Xenopus* heterologous system, using the human orthologues (Beltrán et al., 2013). To validate the use of the mouse as animal model for the effect of piperine (**1a**) on KCNK channels we expressed the mouse orthologues for KCNK3, KCNK9 and KCNK18, whose human counterparts have been shown to be inhibited by piperine, in *Xenopus* oocytes and exposed them to increasing concentrations of piperine (1–1000 μM). Through this approach, we obtained IC_50_ values for piperine on mKCNK3 (30.0 ± 2.8 μM) and mKCNK9 (214.0 ± 60.1 μM) that were similar to those reported for their human orthologues (45 ± 3.9 μM and 133.3 ± 5.9 μM for hKCNK3 and hKCNK9, respectively, Beltrán et al., 2013). However, similar to hKCNK18 (IC50 value of 230.7 ± 105 μM, Beltrán et al., 2013), its mouse orthologue was much less affected by piperine (**Figure 2**). We considered this result as validating the use of the mouse as a model, and as a next step and for a quick access to a large number of cells, we used the calcium imaging technique on primary cultured mouse trigeminal neurons. We exposed primary cultured mouse trigeminal neurons to 100 μM piperine as well as to 10 μM of the pungent TRPV1-agonist capsaicin. As expected, the application of 100 μM piperine induced a calcium response in a subset of trigeminal neurons (68 out of 619 neurons). Interestingly, from the 68 piperine-responsive neurons, we observed that only 35 (51%) were also capsaicin-responsive (**Figure 3A**). To answer the question as to whether a calcium response could still be observed in piperine-responsive neurons after preventing piperine from activating either TRPV1 or TRPA1, we exposed the mouse trigeminal neurons to piperine in the presence of the classical TRPV1 and TRPA1 antagonists capsazepine (5 μM) and HC-030031 (20 μM). Interestingly, 25 of the 37 piperine-responsive neurons (68%) were still responsive when piperine was applied in the presence the TRP antagonists (**Figures 3B,C**). Our results indicated the presence of more than one population of piperine-responsive neurons. We were therefore interested in the distribution of piperine-sensitive neurons in their function to cell diameter and their responsiveness toward the classical TRPV1-agonist capsaicin. The histogram of piperine- and capsaicin-responsive neurons shows a unimodal distribution, with most neurons presenting small cell diameters corresponding to nociceptive neurons associated with the perception of pungency. Interestingly, piperine- as well as capsaicin-sensitive neurons were also found among those of larger cell diameters (**Figure 3D**).

**FIGURE 4:**
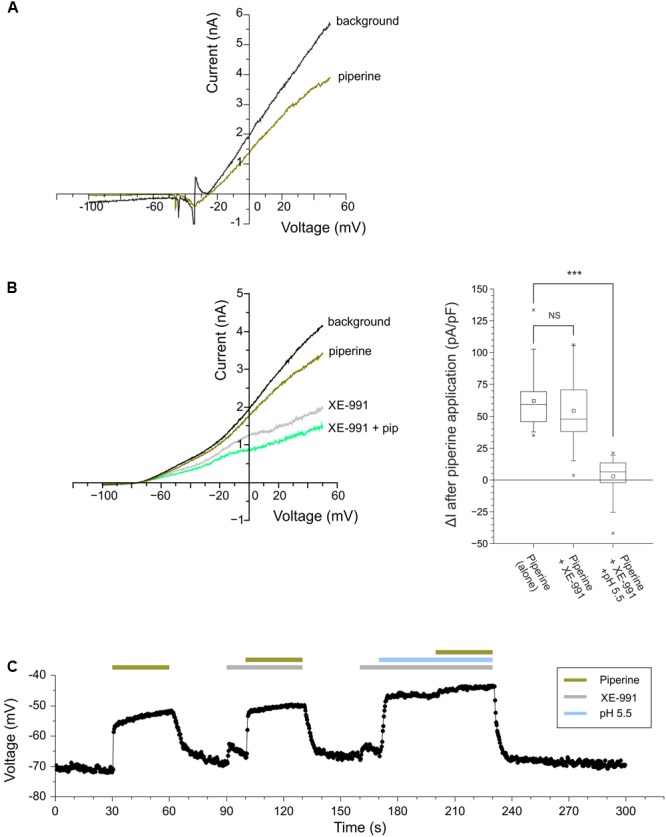
**(A)** Representative current-voltage curves from cultured mouse trigeminal neurons subjected to a voltage ramp from –100 to 50 mV. The traces compare the change in background current after the application of 100 μM piperine under physiological extracellular solution. **(B)** *Left:* A representative current-voltage curve of a cultivated mouse trigeminal neuron under Na^+^/Ca^2+^-free solution before application (black trace), after the application of 100 μM piperine (dark green trace), the KCNQ selective blocker XE-991 (100 μM, gray trace) and subsequent co-application of 100 μM XE-991 and 100 μM piperine (light green trace). *Right*: The comparative effect of piperine (100 μM) alone, co-applied with XE-991 (100 μM) and co-applied with XE-991 under acidic pH. *N* = 11 neurons, mean values are indicated by an open rectangle, box for a range between 25 and 75 percentiles with a line for the median, whiskers for a range between the 5 and 95 percentiles, maximum and minimum value data are indicated with an x. **(C)** A representative current-clamp recording from a cultivated mouse trigeminal neuron exposed to piperine (100 μM), piperine co-applied with XE-991 and piperine co-applied with XE-991 under acidic pH.

These results indicated that piperine was also able to excite trigeminal neurons by a TRP-independent mechanism. Our next step was to investigate whether this mechanism(s) could involve KCNK channels. KCNK channels play a key role in maintaining the resting potential of excitable cells (Enyedi and Czirják, 2010). Previous studies have shown that KCNK inhibitors, e.g., the tingling compound hydroxy-α-sanshool, depolarize trigeminal neurons by decreasing their background K^+^ current (Bautista et al., 2008). We therefore asked whether piperine could induce a similar effect on the background current of mouse trigeminal neurons. For this purpose, we used the whole-cell patch clamp technique and observed that 100 μM piperine was able to induce a decrease in the background current by 19.5 ± 1.8% (N of 13 neurons out of 38 evaluated neurons, all evaluated neurons were of <30 μm diameter, see **Figure 4A**). This effect was also present after replacing extracellular Na^+^ with NMDG, on a nominal Ca^2+^-free extracellular solution containing 5 mM of the calcium chelator EGTA (**Figure 4B**, left). Furthermore, classical K^+^ channel blockers, such as TEA (1 mM) and 4-AP (1 mM), did not hamper the inhibitory effect of piperine (data not shown). We observed that pre-incubation with XE-991, a potent and selective blocker for the voltage-gated KCNQ channels (Yeung and Greenwood, 2005), induced a marked decrease in the background current; however, it failed to prevent the piperine response. Interestingly, when piperine was applied under acidic pH, the current inhibition induced by this compound was significantly decreased (*p* = 0,00012), suggesting members of TASK and/or TREK subfamilies as possible targets (**Figure 4B**, right). In accordance with these observations, current clamp experiments under the same Na^+^/Ca^2+^-free conditions showed that the application of piperine led to a mild depolarization, whose amplitude was not markedly affected by preincubation with XE-991 but was strongly impaired under acidic pH (**Figure 4C**).

**FIGURE 5:**
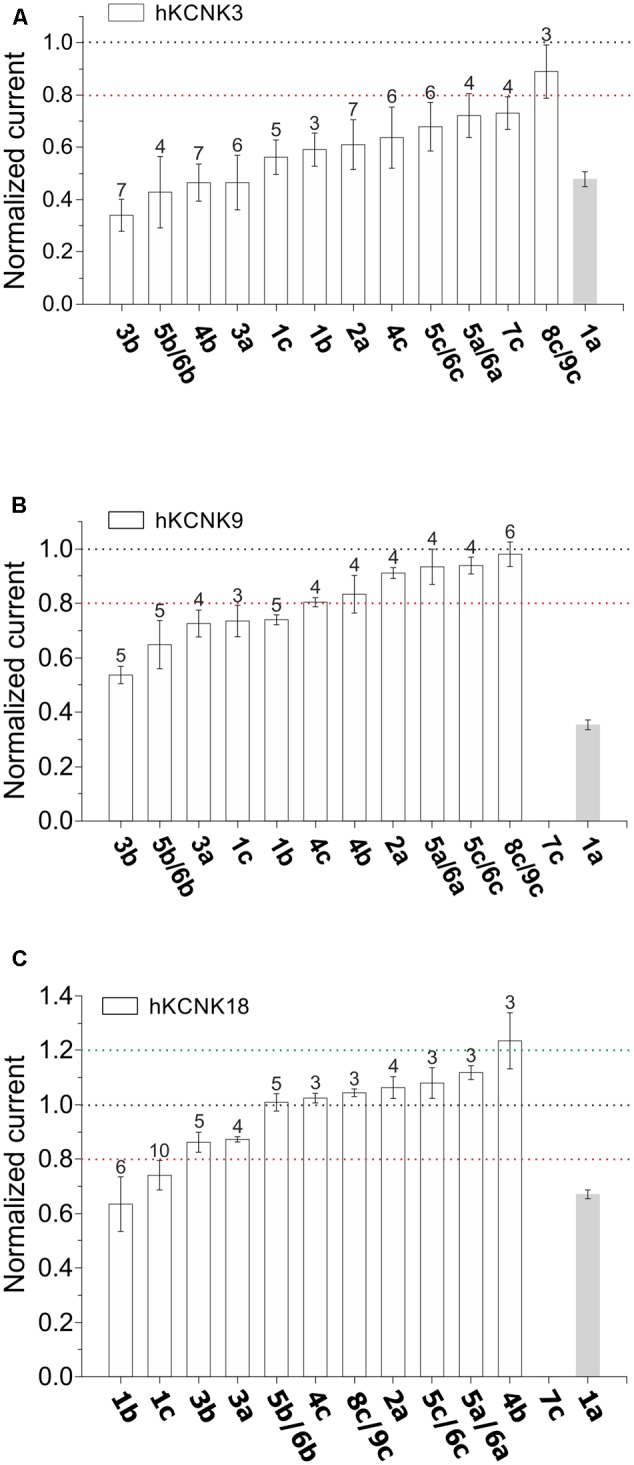
Normalized currents showing the effect of 12 sensory active compounds isolated from black peppercorns (see **Figure 1**) at 1 mM on *Xenopus laevis* oocytes expressing hKCNK3 **(A)**, hKCNK 9 **(B)**, and hKCNK18 **(C)**. *N* = 3–7 oocytes. The currents were normalized to the current registered prior to the application of each substance (dotted black line, see materials and methods section for further details). If a substance was able to induce a change greater than 20% of the basal activity (as indicated by upper and lower dotted lines), the effect was considered relevant. For all graphics, the reported effect of 1 mM piperine is provided as a reference (gray bar).

**FIGURE 6:**
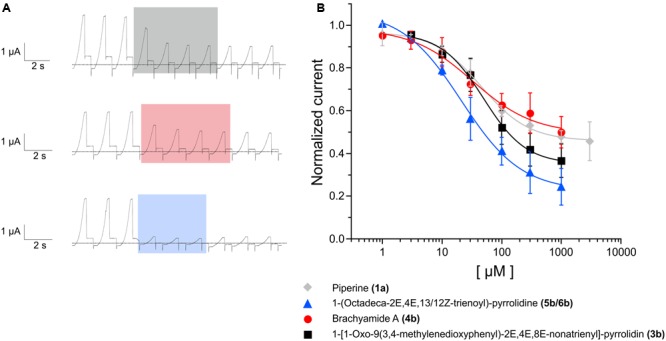
**(A)** Representative voltage-clamp recordings from *Xenopus oocytes* expressing hKCNK3, before, during and after the application of 1 mM of 1-[1-oxo-9(3,4-methylenedioxyphenyl)-2*E*,4*E*,8*E*-nonatrienyl]-pyrrolidine **(3b)** (top); brachyamide A **(4b)** (middle) and 1-(octadeca-2*E*,4*E*,13/12Z-trienoyl)pyrrolidine **(5b/6b)** (bottom). The dotted line represents zero current. **(B)** Dose-response curves for the effect of 1-[1-oxo-9(3,4-methylenedioxyphenyl)-2*E*,4*E*,8*E*-nonatrienyl]-pyrrolidine **(3b)** (black squares); brachyamide A **(4b)** (red circles) and 1-(octadeca-2*E*,4*E*,13/12*Z*-trienoyl)-pyrrolidine **(5a/6a)** (blue triangles) on hKCNK3. See **Table 1** for IC_50_ values. *N* = 3–5 oocytes.

**FIGURE 7:**
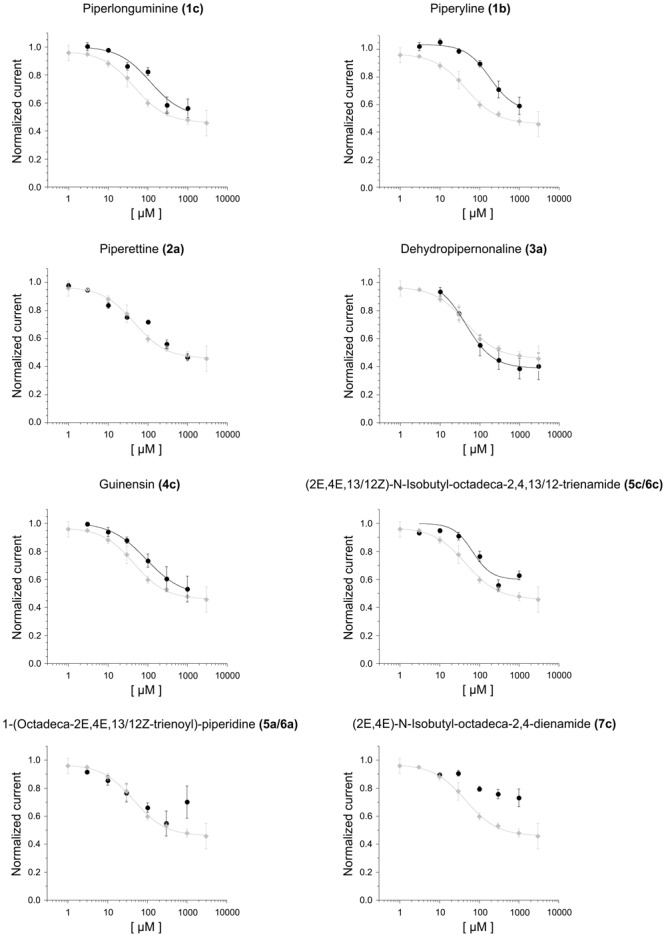
The dose-response relationship for piperlonguminine **(a, 1c)**, piperyline **(b, 1b)**, piperettine **(c, 1c)**, dehydropipernonaline **(d, 3a)**, guineensine **(e, 4c)**, (2*E*,4*E*,13/12*Z*)-*N*-isobutyl-octadeca-2,4,12/13-trienamide **(f, 5c/6c)**, 1-(octadeca*-*2*E*,4*E*,13/12*Z*-trienyl)piperidine **(g, 5a/6a)** and (2*E*,4*E*)-*N*-isobutyl-octadeca-2,4-dienamide **(h, 7c)** on *Xenopus* oocytes expressing hKNCK3. *N* = 3–6 oocytes. The dose-response curve for piperine on hKCNK3 (gray curve) from Beltrán et al., 2013 is presented for comparison purposes.

**FIGURE 8:**
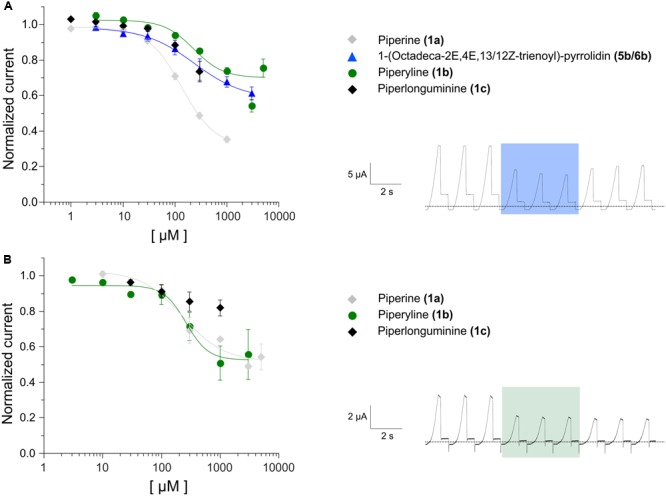
**(A)** *Left*: A representative voltage-clamp recording from a *Xenopus* oocyte expressing hKCNK9, before, during and after the application to 1 mM of 1-(octadeca-2*E*,4*E*,13/12*Z*-trienoyl)pyrrolidine **(5b/6b)**. *Right*: Dose-response curves for the effect of 1-(octadeca-2*E*,4*E*,13/12*Z*-trienoyl) pyrrolidine **(5b/6b)** on hKCNK9. The IC_50_ value is 236 ± 56 μM. *N* = 3–6 oocytes. **(B)** *Left*: A representative voltage-clamp recording from a *Xenopus* oocyte expressing hKCNK18, before, during and after the application to 1 mM of piperyline **(1b)**. *Right*: Dose-response curves for the effect of piperyline **(1b)** on hKCNK18. The IC_50_ value is 252 ± 55 μM. *N* = 3–6 oocytes. The dose-response curve for piperine **(1a)** (gray diamonds) on each channel is presented for comparison.

These piperine results were highly suggestive of a role for KCNK channels in the chemoperception of piperine. Therefore, we asked whether the other sensory active compounds isolated from black peppercorns could also affect the basal activity of human KCNK channels 3, 9, and 18. For this purpose, selected pungent and pungent/tingling substances (**1a**-**9c**; **Figure 1A**) were isolated in purities higher than 98% and identified by means of LC-MS, UPLC-TOF-MS as well as 1/2D NMR experiments. We tested these at a concentration of 1 mM in our cell system (**Figure 5**). Due to the high amounts required for calcium imaging experiments, the study of these compounds was restricted to the *Xenopus* system. Remarkably, the basal activity of hKCNK3 was significantly inhibited by most of the tested compounds, with 1-(octadeca-2*E*,4*E*,13/12*Z*-trienoyl)pyrrolidine (**5b/6b**, pungent and tingling), brachyamide A (**4b**, pungent), and 1-[1-oxo-9(3,4-methylenedioxyphenyl)-2*E*,4*E*,8*E*-nonatrienyl]-pyrrolidine (**3b**, pungent) presenting the most pronounced effect (**Figures 5, 6**). These compounds induced a partial, long-lasting, reversible, and dose-dependent inhibition of hKCNK3 with IC_50_ values of 19 ± 2.1, 32 ± 11, and 44 ± 6.7 μM, respectively (**Figure 6**, see **Table 1** and **Figure 7** for a complete list of IC_50_ values). Additionally, 1-(octadeca-2*E*,4*E*,13/12*Z*-trienoyl)pyrrolidine (**5b/6b**, pungent and tingling) also inhibited hKCNK9, although with far less potency. All three KCNK channels presented a decrease in their basal activity after exposure to piperyline (**1b**) and to a lesser degree, piperloguminine (**1c**), the pyrolidine and isobutylamine analogs of piperine (**1a**) (**Figures 5, 8**), respectively. Moreover, it could be observed that the rank order of compounds no. **1a**-**9c** for the inhibition of human KCNK channel 3, 9 and 18 varies between themselves.

**Table 1:** The effect of chemosensates from black peppercorns on hKCNK3.

Chemosensate (no.^a^)	IC_50_ (in μM)	% of basal activity after saturating concentrations
1-(octadeca-2*E*,4*E*,13/12*Z*-trienoyl)-pyrrolidine **(5b/6b)**	19 ± 2.1	27 ± 3%
brachyamide A **(4b)**	32 ± 11	51 ± 5%
piperine **(1a)** (from Beltrán et al., 2013)	45 ± 3.9	46 ± 10%
dehydropipernonaline **(3a)**	44 ± 6.7	39 ± 1%
1-[1-oxo-9(3,4-methylenedioxyphenyl)-2*E*,4*E*,8*E*-nonatrienyl]-pyrrolidine **(3b)**	49.6 ± 8.5	35 ± 3%
(2*E*,4*E*,13/12*Z*)-*N*-isobutyl-octadeca-2,4,12/13-trienamide **(5c/6c)**	70 ± 32.8	57 ± 7%
guineensine **(4c)**	90.9 ± 12.7	48 ± 2.8%
piperlongumine **(1c)**	113.5 ± 79.5	50 ± 14%
piperyline **(1b)**	180 ± 39	55 ± 5%

***^a^**The structures of the numbered compounds are given in **Figure 1**.*

*A list of IC_50_ values and the percentage of basal activity that remains after saturating concentrations of each chemosensate is achieved.*

## Discussion

Black pepper (*Piper nigrum* L.) has been highly appreciated by consumers all over the world for the typical pungent and tingling orosensory impression elicited by its corns. We addressed the question of whether it was plausible that KCNK channels could constitute a physiologically relevant target for the sensory active compounds present in black peppercorns. Prior research had demonstrated that pungent/tingling-sensing *Zanthoxylum* alkylamides, such as α-hydroxysanshool, which activates TRPV1 and TRPA1 channels (Sugai et al., 2005; Koo et al., 2007; Menozzi-Smarrito et al., 2009; Bader et al., 2014), additionally excite sensory neurons by inhibiting KCNK channels (Bautista et al., 2008). No similar evidence was available for exclusively pungent-sensing compounds on KCNK channels, as our previous experiments were limited to a heterologous system (Beltrán et al., 2013). It was also not clear if they could be involved in the perception of pungency, which is mostly associated with the activation of TRP channels, especially TRPV1 (Ramsey et al., 2006).

Our results concerning the piperine-responsive neuronal subpopulations showed that a high percentage of piperine-responsive neurons were not responsive to the standard TRPV1-agonist capsaicin. Also of interest was that piperine-responsive neurons were also found among those with larger cell bodies, although the majority corresponded to neurons with small cell areas. In addition, the use of selective antagonists for the piperine-induced activation of both TRPV1 and TRPA1 channels (Okumura et al., 2010), failed to prevent a piperine response in 68% of the piperine-responsive trigeminal neurons. These observations indicate the existence of a piperine-responsive subpopulation whose response to piperine does not require TRP-activation. This, in turn, suggests a more complex mechanism for piperine chemoperception. It could be speculated that apart from the difference in the potency of these two substances on a given receptor, i.e., TRPV1, the difference in the impression that piperine and capsaicin elicit would rather be due to the different subsets of trigeminal neurons they excite. However, it is worth mentioning that, as previously shown (Liu and Simon, 1996), there is also a considerable overlap between piperine- and capsaicin-sensitive neurons.

With regard to the mechanism by which piperine causes neuronal activation, our whole-cell patch-clamp experiments in mouse trigeminal neurons showed that piperine induces a decrease in the background K^+^ current. This current plays a key role in maintaining the membrane resting potential closer to the K^+^ reversal potential, thus preventing depolarization. Interestingly, the piperine-induced decrease in the background K^+^ current was significantly lower when piperine was applied under acidic pH, but not when co-applied with TEA, 4-AP or XE-991. This would suggest the involvement of KCNK channels of the TASK (KCNK3, KCNK9) or TREK (KCNK2, KCNK4, and KCNK10) subfamilies. However, we previously showed that members of the TREK subfamily, contrary to TASK and TRESK (hKCNK18) members, are not significantly affected by piperine (Beltrán et al., 2013), leaving TASK channels as a most likely target for piperine. Further experiments, preferably with neurons from animals lacking TASK channels, together with more precise studies of the pH influence on the piperine-induced inhibition, could help determine with certainty the role these ion channels may play in the chemoperception of piperine

Our results also demonstrated that, in addition to piperine, several pungent and tingling compounds from *Piper nigrum* possess an inhibitory effect on hKCNK channels, and similar to piperine, their effect is more pronounced on hKCNK3. Interestingly, we observed that piperloguminine (**1c**) as well as piperyline (**1b**), two compounds with low thresholds for oral pungency (0.01 and 0.005 μmol/cm^2^), inhibit all three KCNK channels evaluated. In addition, there was a relative long-lasting effect of most of these compounds on KCNK channels, which contrasts with their reported rapid desensitization on TRPV1 yet correlates with the long-lasting sensation they elicit. Interestingly, none of the above-mentioned substances exerted a complete inhibition on any of the three studied channels. These data expand the relatively sparse knowledge about the pharmacology of KCNK channels and presents 1-(octadeca-2*E*,4*E*,13/12*Z*-trienoyl)pyrrolidine (**5b/6b**) as one of the most potent naturally occurring inhibitors for hKCNK3. Further studies with the isolated chemosensates on mouse trigeminal neurons will help confirm the physiological relevance of these interactions.

Based on their potency to elicit a pungent or/and tingling sensation, it is already known that piperidine analogs in *Piper nigrum* L. always showed lower thresholds *in vivo* when compared to the isobutylamine and the pyrrolidine derivatives (Dawid et al., 2012). This trend could also be observed in our cell-based receptor expression assay. For example, the IC_50_ value obtained for piperine *in vitro* (**1a**, 42 μM) was three times lower compared with those of piperlongumine (**1c**, 113.5 μM) or piperyline (**1b**, 180 μM). In addition, 1-(octadeca-2*E*,4*E*,13/12*Z*-trienoyl)pyrrolidine (**5b/6b**), which shows a conjugated 2,4-dienoic acid amide structure with an additional *cis*-configured double bound and induces both a pungent as well as a long-lasting tingling effect in human sensory tests, had the lowest IC_50_ value and percentage of basal activity on hKCNK3 (**Table 1**).

It is important to mention that our results do not exclude TRP channels as targets for chemosensates from black peppercorns, as both families of ion channels might have a complementary role, since neither of them alone is sufficient to fully understand the mechanism by which black peppercorns elicit their characteristic impression. Current research has recently shown that some members of the KCNK family “fine-tune” the cellular response to stimuli that activate TRP channels, e.g., temperature (Noël et al., 2009). Addtionally, a complementary role of KCNK channels will help to explain the apparent mismatch between the pharmacological data obtained from heterologously expressed TRP channels and the sensomics data. It is worth mentioning that in rat trigeminal neurons, KCNK channels of the TREK subfamily, in particular rKCNK2, have been shown to be co-expressed with TRP channels in the same neuron (Yamamoto et al., 2009). To study the possible co-expression of TASK channels and TRP channels, such as TRPV1 and/or TRPA1, will therefore be of interest. Finally, although our results indicate that KCNK channels are most likely to play a role in the chemoperception of sensory active compounds from black peppercorns, further studies, preferentially with mice lacking KCNK3/KCNK9, are required to fully elucidate the precise function of these channels.

## Author Contributions

Conceived and designed the experiments: CD, LB, HH, TH, GG. Performed the experiments: LB, CD, MB, STi, VP, MS, JL, STh. Analyzed the data: CD, LB. Wrote the paper: LB, CD. LB and CD contributed equally to the present work.

## Conflict of Interest Statement

The authors declare that the research was conducted in the absence of any commercial or financial relationships that could be construed as a potential conflict of interest.
